# Risk factor analysis for cisplatin-induced nephrotoxicity with the short hydration method in diabetic patients

**DOI:** 10.1038/s41598-023-44477-w

**Published:** 2023-10-10

**Authors:** Yoshitaka Saito, Masaki Kobayashi, Shinya Tamaki, Katsuyuki Nakamura, Daisuke Hirate, Kenta Takahashi, Yoh Takekuma, Jun Sakakibara-Konishi, Yasushi Shimizu, Ichiro Kinoshita, Mitsuru Sugawara

**Affiliations:** 1 Department of Clinical Pharmaceutics & Therapeutics, Faculty of Pharmaceutical Sciences, Hokkaido University of Science, 4-1, Maeda 7-Jo 15-Chome, Teine-Ku, Sapporo, 006-8585 Japan; 2 Department of Pharmacy, Hokkaido University Hospital, Kita 14-Jo, Nishi 5-Chome, Kita-Ku, Sapporo, 060-8648 Japan; 3 Laboratory of Clinical Pharmaceutics & Therapeutics, Faculty of Pharmaceutical Sciences, Hokkaido University, Kita 12-Jo, Nishi 6-Chome, Kita-Ku, Sapporo, 060-0812 Japan; 4 Department of Pharmacy, KKR Sapporo Medical Center, 3-40, Hiragishi 1-Jo 6-Chome, Toyohira-Ku, Sapporo, 062-0931 Japan; 5 Division of Hospital Pharmacy, Sapporo Medical University, 291, Minami 1-Jo, Nishi 16-Chome, Chuo-Ku, Sapporo, 060-8543 Japan; 6 Department of Pharmacy, Teine Keijinkai Hospital, 1-40, Maeda 1-Jo 12-Chome, Teine-Ku, Sapporo, 006-8555 Japan; 7 Department of Pharmacy, NTT Medical Center Sapporo, Minami 1-Jo, Nishi 15-Chome, Chuo-Ku, Sapporo, 060-0061 Japan; 8 Department of Respiratory Medicine, Faculty of Medicine, Hokkaido University, Kita 15-Jo, Nishi 7-Chome, Kita-Ku, Sapporo, 060-8638 Japan; 9 Department of Medical Oncology, Faculty of Medicine and Graduate School of Medicine, Hokkaido University, Kita 15-Jo, Nishi 7-Chome, Kita-Ku, Sapporo, 060-8638 Japan; 10 Laboratory of Pharmacokinetics, Faculty of Pharmaceutical Sciences, Hokkaido University, Kita 12-Jo, Nishi 6-Chome, Kita-Ku, Sapporo, 060-0812 Japan

**Keywords:** Chemotherapy, Lung cancer, Cancer, Nephrology

## Abstract

The occurrence of cisplatin (CDDP)-induced nephrotoxicity (CIN) has decreased with advancements in supportive care. In contrast, we reported that baseline diabetes mellitus (DM) complications significantly worsen CIN. This study aimed to determine further risk factors associated with CIN development in DM patients. Patients with thoracic cancer requiring DM pharmacotherapy, who received CDDP (≥ 60 mg/m^2^)-containing regimens using the short hydration method (n = 140), were enrolled in this retrospective multicenter observational study. The primary endpoint of the present study was the elucidation of risk factors (patient factors, DM medication influence, and treatment-related factors) associated with CIN development in patients with DM. Cisplatin-induced nephrotoxicity occurred in 22.1% of patients with DM. The median worst variation of serum creatinine levels and creatinine clearance (worst level − baseline level) was 0.16 mg/dL (range: − 0.12–1.41 mg/dL) and − 15.9 mL/min (− 85.5–24.3 mL/min), respectively. Multivariate logistic regression analyses identified female sex as the singular risk factor for CIN development in the DM population (adjusted odds ratio; 2.87, 95% confidence interval; 1.08–7.67, *P* = 0.04). Diabetes mellitus medication and treatment-related factors did not affect CIN development. In conclusion, our study revealed that female sex is significantly associated with CIN development in patients with DM and thoracic cancer.

## Introduction

Chemotherapy is the main treatment for advanced cancer, and the management of chemotherapy-induced adverse effects is one of the most important factors in providing more effective treatment and maintaining the quality of life of patients^1^.

Cisplatin (CDDP) is a key chemotherapeutic agent used to treat lung, head and neck, ovarian, esophageal, and urological malignancies^2^. Cisplatin-induced nephrotoxicity (CIN) is known to be its dose-limiting toxicity^2^, and reportedly occurs in 5–40% of the patients^2–4^. It is usually reversible, but becomes unreversible in cases of severe symptoms, and symptomatic treatment is limited^2–4^. Oxidative stress, DNA damage, mitochondrial dysfunction, inhibition of protein synthesis, involvement of the tumor necrosis factor family, and decreased autophagy are all associated with CIN development^3–8^.

Magnesium supplementation, quality antiemetic therapy, and appropriate diuretic and hydration administration are the most important CIN prophylaxes and contribute to the reduction of CIN development to 0–10%^3,4^. Many reports evaluated CIN risk factors; however, most of the reports were not performed following the most recent CDDP administration methods with sufficient CIN prophylaxis described above^4^. It has been reported that the co-administration of non-steroidal anti-inflammatory drugs (NSAIDs) and baseline comorbidities of diabetes mellitus (DM) are risk factors for CIN development in the short hydration method, which is the most advanced method for CDDP administration^3^. Non-steroidal anti-inflammatory drugs have already been reported to worsen CIN^9–11^. In addition, it has been shown that baseline DM complications significantly increase the CIN development rate in the method^4^. The prevalence of DM and prediabetes is growing^12^. Therefore, opportunities for administering CDDP to patients with DM are expected to increase.

This study aimed to determine the risk factors associated with CIN development in a short hydration method in patients with DM for early treatment.

### Ethics approval and consent to participate

All procedures performed in this study were conducted in accordance with the ethical standards of the institutional and national research committee and the 1964 Helsinki Declaration and its later amendments, or comparable ethical standards. This study was approved by the institutional review board of each participating institution (in case of Hokkaido University Hospital, approval number: 020–0366). The requirement for formal consent for this study was waived by the Ethical Review Board for Life Science and Medical Research of Hokkaido University Hospital and the Institutional Review Boards of KKR Sapporo Medical Center, Sapporo Medical University, Teine Keijinkai Hospital, and NTT Medical Center Sapporo.

## Results

### Patient characteristics

**Figure 1:**
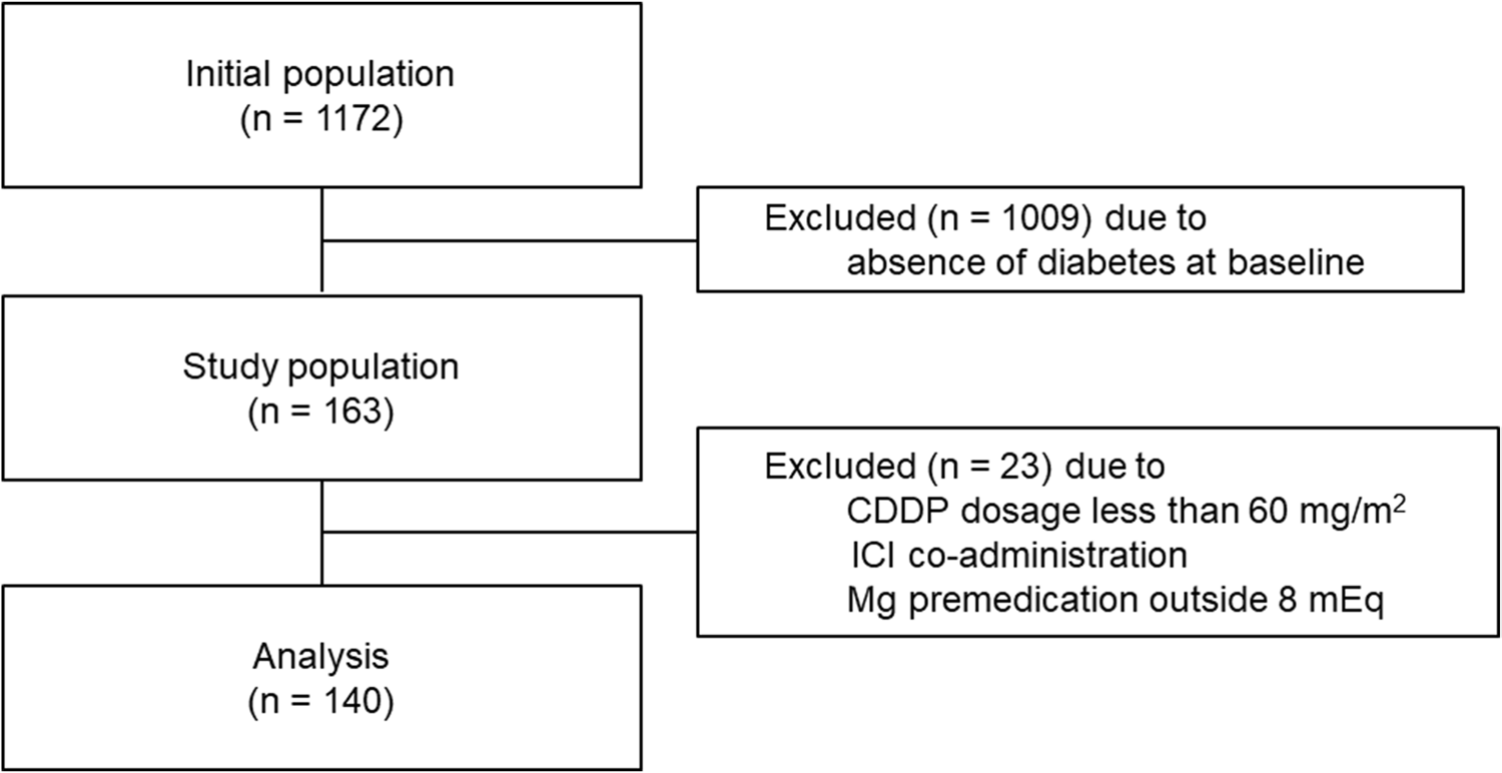
Consort diagram of this study. *CDDP* cisplatin, *ICI* immune-checkpoint inhibitors, *Mg* magnesium.

In total, 140 patients with thoracic cancer who underwent DM pharmacotherapy and received CDDP-containing treatment (≥ 60 mg/m^2^) were enrolled based on the eligibility criteria of this retrospective multicenter observational study (Fig. 1). Baseline patient characteristics are shown in Table 1. Approximately 84% of the participants were males, and the median age was 67 years (range 46–76 years). The percentage of patients with advanced cancer was 35.7% and 11.4% of those had undergone prior treatment. The proportion of patients who received chemoradiotherapy was 24.3%. Median baseline serum albumin level was 4.0 g/dL (2.2–5.0 g/dL), and that of HbA1c l was 7.0% (5.4–11.1%). Median baseline creatinine clearance was 93.7 mL/min (48.8–176.6 mL/min), and 2.9% of participants had less than 60 mL/min. The median CDDP dose in the first cycle was 80 mg/m^2^, and 2.9% of the participants received a dose reduction from treatment initiation. Patients who received co-administration of proton pump inhibitors (PPIs) or NSAIDs during treatment accounted for 36.4% and 20.7%, respectively. Dipeptidyl peptidase-4 (DPP-4) inhibitors or glucagon-like peptide-1 (GLP-1) analogs were administered to 75.7% of patients, followed by metformin (46.4%), sulfonylurea agents or glinides (32.9%), insulin (25.0%), sodium-glucose cotransporter 2 inhibitors (13.6%), and α-glucosidase inhibitors (10.0%). The total number of treatment cycles was 4 for 49.3%, one or two for 18.6%, 3 for 9.3%, and 5 or 6 for 2.1% each. In addition, 30% of the patients received a dose reduction during treatment because of gastrointestinal symptoms, neutropenia, febrile neutropenia, and fatigue; however, no patient received a reduction because of CIN.

**Table 1:** Patient characteristics.

No. of patients	140
Sex: male/female (n)	117/23
Median age (range)	67 (46–76)
Performance status (ECOG) (n, %)	
0/1	134 (95.7%)
2	6 (4.3%)
BSA (m^2^) (median, range)	1.73 (1.36–2.25)
Staging (n, %)	
Stage IV/recurrence	50 (35.7%)
Others	90 (64.3%)
Histology (n, %)	
Adeno	55 (39.3%)
Small	45 (32.1%)
Squamous	22 (15.7%)
Others	18 (12.9%)
Bone metastasis (n, %)	17 (12.1%)
Liver metastasis (n, %)	9 (6.4%)
Brain metastasis (n, %)	14 (10.0%)
Treatment line (n, %)	
First line	124 (88.6%)
Second or later line	16 (11.4%)
Adjuvant chemotherapy setting (n, %)	45 (32.1%)
Radiation combination (n, %)	34 (24.3%)
Albumin (g/dL) (median, range)	4.0 (2.2–5.0)
Hemoglobin (g/dL) (median, range)	13.0 (8.2–16.7)
HbA1c (%) (median, range)	7.0 (5.4–11.1)
Liver dysfunction (n, %)	25 (17.9%)
Creatinine (mg/dL) (median, range)	0.69 (0.35–1.15)
CCr (mL/min) (median, range)	93.7 (48.8–176.6)
< 60 mL/min (n, %)	4 (2.9%)
Sodium (mEq/L) (median, range)	139 (128–145)
Potassium (mEq/L) (median, range)	4.2 (3.0–5.6)
Chloride (mEq/L) (median, range)	104 (94–110)
Calcium (mg/dL) (median, range)	9.4 (8.0–10.7)
Hypertension (n, %)	68 (48.6%)
Smoking history (Former or Current) (n, %)	133 (95.0%)
Current smoker	53 (37.9%)
Alcohol intake (≥ 5 days in a week) (n, %)	73 (52.1%)
Chemotherapy regimen (n, %)	
CDDP + VNR	54 (38.6%)
CDDP + ETP	41 (29.3%)
CDDP + PEM	22 (15.7%)
CDDP + IRI	11 (7.9%)
CDDP + PEM + BV	10 (7.1%)
CDDP + GEM	2 (1.4%)
CDDP dosage (mg/m^2^) (n, %)	
80	93 (67.9%)
75	32 (21.4%)
64	4 (2.9%)
60	11 (7.9%)
Co-administration of PPIs (n, %)	51 (36.4%)
Co-administration of NSAIDs (n, %)	29 (20.7%)
Types of diabetes medication	
DPP-4 inhibitors or GLP-1 analogs	106 (75.7%)
Metformin	65 (46.4%)
Sulfonylurea agents or glinides	46 (32.9%)
Insulin	35 (25.0%)
Sodium-glucose cotransporter 2 inhibitors	19 (13.6%)
α-glucosidase inhibitors	14 (10.0%)
Pioglitazone	0 (0%)

*ECOG* Eastern Cooperative Oncology Group, *BSA* body surface area, *CCr* creatinine clearance, *CDDP* cisplatin, *VNR* vinorelbine, *ETP* etoposide, *PEM* pemetrexed, *IRI* irinotecan, *BV* bevacizumab, *GEM* gemcitabine, *PPIs* proton pump inhibitors, *NSAIDs* non-steroidal anti-inflammatory drugs, *DPP-4* dipeptidyl peptidase-4, *GLP-1* glucagon-like peptide-1.

Liver dysfunction: Grade 1 or higher aspartate aminotransferase, alanine aminotransferase, and total bilirubin levels.

The types of medications include duplicates.

### Development of CIN and variation of serum creatinine levels and creatinine clearance

CIN was confirmed in 22.1% of patients. Median worst variation of serum creatinine (SCr) levels and creatinine clearance (worst level − baseline level) was 0.16 mg/dL (range: − 0.12–1.41 mg/dL) and − 15.9 mL/min (− 85.5–24.3 mL/min), respectively (Table 2). Most cases of CIN (80.6%) were confirmed during the early course of treatment, and no patient newly experienced CIN after the fifth cycle. The worst creatinine clearance was also confirmed during the early course of treatment. Moreover, CIN at the final evaluation (approximately 3 weeks after the last CDDP administration), which can be associated with unreversible CIN, was confirmed in 10.0% of the participants.

**Table 2:** Development of CIN and variation of serum creatinine levels and creatinine clearance.

Development of CIN (n, %)	
Worst evaluation	31 (22.1%)
Final evaluation	14 (10.0%)
Serum creatinine levels (mg/dL) (median, range)	
Baseline levels	0.69 (0.35–1.15)
Worst levels	0.87 (0.53–2.02)
Final levels	0.76 (0.39–1.42)
Worst variation levels	0.16 (− 0.12–1.41)
Final variation levels	0.08 (− 0.22–0.65)
Creatinine clearance (mL/min) (median, range)	
Baseline levels	93.7 (48.8–176.6)
Worst levels	73.9 (32.3–145.0)
Variation levels	− 15.9 (− 85.5–24.3)
Cycle of CIN incidence	
1	21 (67.7%)
2	4 (12.9%)
3	4 (12.9%)
4	2 (6.5%)
Cycle of worst creatinine clearance	
1	74 (52.9%)
2	26 (18.6%)
3	17 (12.1%)
4 or later	23 (16.4%)

*CIN* cisplatin-induced nephrotoxicity.

Worst evaluation: variation between worst and baseline levels.

Final evaluation: variation between final (approximately 3 weeks after final cisplatin administration) and baseline levels.

### Risk factor analysis for CIN development

Univariate and multivariate logistic regression analyses were performed to identify the independent risk factors for CIN development in patients with DM (Table 3). Multivariate analysis identified that female sex was the singular independent patient’s risk factor (adjusted odds ratio; 2.87, 95% confidence interval; 1.08–7.67, *P* = 0.04). However, DM medication or CDDP-including treatment-related factors did not affect CIN development. Notably, the co-administration of NSAIDs, suggested factors in all patient populations in a meta-analysis^11^, was not suggested as a factor. The association between chemotherapy-induced gastrointestinal symptoms and CIN development was also assessed, as oral hydration plays an important role in the short hydration method. Patients exhibiting all-grade nausea, vomiting, and anorexia accounted for 58.6, 5.0, and 65.0% of the patients, respectively. Concerning problematic grade ≥ 2 symptoms that can affect oral hydration, the values were 20.7, 1.4, and 27.9%, respectively. However, its development was not associated with the development of CIN.

**Table 3:** Univariate and multivariate analyses of the risk factors associated with the incidence of CIN in DM patients.

	Univariate analysis		Multivariate analysis	
	Odds ratio (95%CI)	*P*-value	Odds ratio (95%CI)	*P*-value
(A) Patient factor				
Sex				
Female/male	2.78 (1.07–7.23)	0.04*	2.87 (1.08–7.67)	0.04*
Age (years)				
≥ 65/< 65	0.77 (0.34–1.74)	0.53	Excluded	–
Performance status (ECOG)				
2/0 or 1	1.81 (0.32–10.38)	0.51	Excluded	–
Staging				
Stage IV or recurrence/others	0.82 (0.35–1.92)	0.65	Excluded	–
BSA (m^2^)				
≥ 1.7/< 1.7	1.25 (0.55–2.82)	0.60	Excluded	–
Anemia				
Present/absent	1.46 (0.65–3.28)	0.36	Excluded	–
Hypoalbuminemia				
Present/absent	1.66 (0.73–3.79)	0.23	Excluded	–
Liver dysfunction				
Present/absent	0.86 (0.29–2.50)	0.78	Excluded	–
Renal dysfunction				
Present/absent	1.18 (0.12–11.74)	0.89	Excluded	–
Hypertension				
Present/absent	1.87 (0.83–4.22)	0.13	1.72 (0.74–3.96)	0.21
Co-administration of PPIs				
Present/absent	1.13 (0.50–2.58)	0.76	Excluded	–
Co-administration of NSAIDs				
Present/absent	1.15 (0.44–3.02)	0.77	Excluded	–
(B) DM medication				
DPP-4 inhibitors or GLP-1 analogs				
Present/absent	1.13 (0.44–2.91)	0.80	Excluded	–
Metformin				
Present/absent	1.31 (0.59–2.90)	0.51	Excluded	–
Sulfonylurea agents or glinides				
Present/absent	0.97 (0.41–2.26)	0.94	Excluded	–
Insulin				
Present/absent	1.60 (0.67–3.84)	0.29	Excluded	–
Sodium-glucose cotransporter 2 inhibitors				
Present/absent	0.62 (0.17–2.29)	0.48	Excluded	–
α-Glucosidase inhibitors				
Present/absent	0.56 (0.12–2.64)	0.46	Excluded	–
(C) Treatment-related factor				
Cisplatin dosage				
> 60 mg/m^2^/60 mg/m^2^	0.46 (0.13–1.70)	0.25	Excluded	–
Treatment courses				
3 or more/2 or less	0.55 (0.24–1.23)	0.14	0.51 (0.22–1.18)	0.11
Dose reduction during the treatment				
Present/absent	1.15 (0.49–2.70)	0.76	Excluded	–
Radiation combination				
Present/absent	1.69 (0.70–4.06)	0.24	Excluded	–
Grade ≥ 2 nausea				
Present/absent	1.46 (0.57–3.71)	0.43	Excluded	–
Grade ≥ 2 vomiting				
Present/absent	3.60 (0.22–59.27)	0.37	Excluded	–
Grade ≥ 2 anorexia				
Present/absent	1.08 (0.45–2.60)	0.87	Excluded	–

*P < 0.05.

*ECOG* Eastern Cooperative Oncology Group, *BSA* Body surface area, *PPIs* proton pump inhibitors, *NSAIDs* nonsteroidal anti-inflammatory drugs, *DM* diabetes mellitus, *DPP-4* dipeptidyl peptidase-4, *GLP-1* glucagon-like peptide-1.

Considering the cutoff at our facility, serum albumin levels less than 4.1 g/dL were defined as hypoalbuminemia.

Liver dysfunction: grade 1 or higher aspartate aminotransferase, alanine aminotransferase, and total bilirubin levels.

Renal dysfunction: creatinine clearance of less than 60 mL/min.

## Discussion

We previously reported that patients with DM develop CIN at a significantly higher rate in a short hydration method^4^. Autophagy reportedly protects against CIN^8,13,14^, and its induction at the proximal tubule, where CIN mostly appears, is significantly suppressed in type 2 DM, which all participants met in previous and present studies^4,15^. Thus, we consider that the main mechanism of CIN degradation in type 2 DM is the reduction in renal autophagy. Owing to advances in supportive care, the development rate of CIN has significantly decreased; however, approximately 30% of patients with DM developed symptoms in our previous study^4^. Consequently, we aimed to identify further risk factors for the early detection and treatment of CIN in patients with DM.

The development rate of CIN was 22.1%, and female sex was identified as the single most significant risk factor for CIN development in patients with DM. Previous studies have reported that women are at a higher risk of developing CIN^9,16–18^. One possible hypothesis is sex differences in autophagy^19,20^. Tao et al. reported that lower visceral adiposity in female mice arises from more active estradiol-estrogen receptor α signaling, which regulates autophagy and adipogenesis^20^. However, sex was not associated with CIN development in the entire patient population in our previous studies^3,4^. In previous reports reporting the results, a short hydration method was not used, and the evaluated CDDP dosage was different from ours^3,4,9,16–18^, which might have induced the incongruence in the results. In addition, as autophagic activity at the proximal tubule is considered to be suppressed in patients with type 2 DM, further suppression in females may have affected the results. However, as the detailed mechanism for this finding remains elusive, further studies are required.

In contrast, the co-administration of NSAIDs was not associated with CIN development in patients with DM. In our previous studies evaluating the risk factors or meta-analyses of CIN in an all-patient population, NSAIDs co-administration was significantly associated with its development^3,11^. The main mechanism of NSAID-induced nephrotoxicity is a decrease in prostaglandin levels owing to cyclooxygenase-1 inhibition^21^. In contrast, prostaglandin E_2_ (PGE_2_) signaling may contribute to the progression and development of type 2 DM with insulin resistance, elevated fasting glucose, and glucose intolerance^22^. Fenske et al. reported that plasma PGE_2_ metabolite levels are twice as high in patients with type 2 DM than in controls^22^. In addition, we have reported that most NSAIDs do not affect renal autophagy in vitro^14^. Thus, we believe that the higher baseline PGE_2_ levels in patients with DM compensate for the renal influence of NSAIDs. However, it is necessary to compare the effects of concomitant NSAIDs use on CIN development between patients with and without DM complications, including PGE_2_ levels, to understand this phenomenon fully.

Autophagy is nephroprotective against CIN^8,13,14^, and autophagic activity is induced in type 1 DM and suppressed in type 2 DM^15^. Furthermore, renal damage caused by CDDP was reportedly reduced in streptozotocin-administered mice, which are recognized as models of type 1 DM^23,24^. The effect of autophagy can differ between DM types; therefore, it is necessary to evaluate the impact of the DM type on CIN clinically, focusing on autophagy.

Managing chemotherapy-induced nausea and vomiting (CINV) is important for sufficient oral hydration. Thus, adequate prevention and appropriate management of breakthrough gastrointestinal symptoms in accordance with the current guidelines are essential. In this study, adequate CINV management, considering CIN, was performed, resulting in no association between adverse gastrointestinal effects and CIN. Consequently, we should try to manage CINV with adequate understanding from the medical team.

This study had some limitations. First, this study was retrospectively conducted in a relatively small population, as patients with DM were limited to 14% of the total population. Second, as all patients in the present study had type 2 DM and renal pathology is different between type 1 and 2 DM^15^, the present results may differ from those of patients with type 1 DM. Third, we excluded patients receiving immune-checkpoint inhibitors (ICIs) as we tried to evaluate the direct association between the evaluated factors and CIN development. However, because combination treatment with ICIs is one of the main treatment strategies for several types of cancer, an assessment of this latter population is needed. Finally, we did not evaluate the genetic backgrounds or autophagy levels of the patients. Organic cation transporter 2 (OCT2), which transports CDDP to the proximal tubule, has single-nucleotide polymorphisms (SNPs)^25^. In addition, the 808G>T SNP in *OCT2* was shown to ameliorate CIN without altering its disposition^26^. Therefore, the patients’ genetic backgrounds and/or other unknown factors may have affected the results. Furthermore, as we consider that autophagy plays an important role in the development of CIN in patients with DM, its assessment is required. Considering these limitations, our preliminary results should be validated by future studies.

In conclusion, our study revealed that female sex is significantly associated with CIN development in a short hydration method in patients with DM and thoracic cancer. Management of high-risk populations is crucial; therefore, further evaluation, particularly regarding prophylaxis, is necessary.

## Methods

### Patients

Patients with thoracic cancer who required DM pharmacotherapy and received CDDP-containing treatment (≥ 60 mg/m^2^) between December 2012 and September 2022 were enrolled. CDDP-including regimens were CDDP (75 mg/m^2^, day 1) + pemetrexed (500 mg/m^2^, day 1) ± bevacizumab (15 mg/kg, day 1), CDDP (80 mg/m^2^, day 1) + vinorelbine (20–25 mg/m^2^, days 1, 8) ± radiation, CDDP (80 mg/m^2^, day 1) + etoposide (100 mg/m^2^, days 1–3) ± radiation, CDDP (60 mg/m^2^, day 1) + irinotecan (60 mg/m^2^, days 1, 8, 15), and CDDP (80 mg/m^2^, day 1) + gemcitabine (1000 mg/m^2^, days 1 and 8).

All patients met the following baseline criteria: (1) age ≥ 20 years; (2) CDDP administration by a short hydration method; (3) 0 to 2 Eastern Cooperative Oncology Group performance status (ECOG-PS); (4) detailed patient information available from medical records; and (5) sufficient renal or liver function for treatment induction. Patients who received ICI co-administration, were transferred to the hospital during chemotherapy, discontinued the treatment during the first cycle, and whose dosage of magnesium premedication was outside 8 mEq were excluded. The primary endpoint of the present study was the elucidation of risk factors (patient factors, DM medication influence, and treatment-related factors) associated with CIN development in patients with DM. Based on our previous report that found CIN in 27% patients with DM^4^, we decided to include approximately 3–4 covariates in the multivariate analysis, resulting in the necessity of enrolling approximately 150 participants.

This study was approved by the Ethical Review Board for Life Science and Medical Research of Hokkaido University Hospital (Approval Number: 020-0515) and the Institutional Review Boards of KKR Sapporo Medical Center, Sapporo Medical University, Teine Keijinkai Hospital, and NTT Medical Center Sapporo, and was conducted in accordance with the Declaration of Helsinki and the STROBE statement. The requirement for formal consent for this study was waived by the Ethical Review Board for Life Science and Medical Research of Hokkaido University Hospital and the Institutional Review Boards of KKR Sapporo Medical Center, Sapporo Medical University, Teine Keijinkai Hospital, and NTT Medical Center Sapporo.

### Treatment methods

The short hydration method has been described in detail in previous reports^3,27,28^. All regimens basically included antiemetic therapy according to the current national guidelines^29^; palonosetron 0.75 mg on day 1, oral aprepitant 125 mg on day 1 and 80 mg on days 2 and 3 or intravenous fosaprepitant 150 mg on day 1, and dexamethasone 9.9 mg infusion on day 1 and 8 mg orally on days 2–4. These prophylactic medications and other additional antiemetic drugs were administered at the physicians’ discretion.

### Evaluation of CIN and other adverse effects

Toxicities in all subsequent treatment cycles were graded according to the Common Terminology Criteria for Adverse Events (CTCAE), version 5.0. Renal function was evaluated based on SCr elevation measured using an enzymatic method. In this study, CIN was defined as a SCr elevation of ≥ 1.5 times or > 0.3 mg/dL from the baseline levels as referenced in previous reports^4,9,10,30^.

### Statistical analysis

Univariate and multivariate logistic regression analyses were performed using the following possible covariates: sex, age, ECOG-PS, staging, body surface area (BSA), anemia, hypoalbuminemia, liver dysfunction (grade 1 or higher aspartate aminotransferase, alanine aminotransferase, total bilirubin elevation), renal dysfunction (creatinine clearance calculated by Cockroft–Gault formula of < 60 mL/min), concomitant hypertension, and co-administration of PPIs and NSAIDs as baseline patient factors; administration of DPP-4 inhibitors or GLP-1 analogs, metformin, sulfonylurea agents or glinides, insulin, sodium-glucose cotransporter 2 inhibitors, and α-glucosidase inhibitors as DM medication influence; and CDDP dosage, treatment courses, dose reduction during the treatment, radiation combination, and development of grade ≥ 2 nausea, vomiting, and anorexia for treatment-related factors according to previous reports^3,4,9,11,16–18,30–38^. Previously reported variables that demonstrated potential associations with CIN development in univariate logistic regression analysis (*P* < 0.20) were considered when building the multivariable model.

All analyses were performed using the JMP statistical software version 16.1 (SAS Institute Japan, Tokyo, Japan). *P*-values less than 0.05 were considered statistically significant.

## Data Availability

The datasets used and/or analyzed in the current study are available from the corresponding author upon reasonable request.
